# Intra- and inter-rater reliabilities of skin mechanical properties measured in healthy individuals using skin elasticity meter

**DOI:** 10.1080/07853890.2023.2279747

**Published:** 2023-11-15

**Authors:** Yudai Fujimoto, Yoshimi Yuri, Yuji Kato, Shota Kinoshita, Hironari Tamiya

**Affiliations:** a Graduate School of Health Sciences, Morinomiya University of Medical Sciences, Osaka, Japan; b Department of Rehabilitation, Osaka International Cancer Institute, Osaka, Japan; c Department of Orthopaedic surgery (Musculoskeletal Oncology Service), Osaka International Cancer Institute, Osaka, Japan

**Keywords:** Skin elasticity meter, skin mechanical properties, reliability, Bland–Altman analysis, measurement, outcomes

## Abstract

**Purpose:**

The aim of this study is to establish a standardized measurement method and to examine the intra- and inter-reliabilities and absolute reliability of measuring skin mechanical properties using a skin elasticity meter (Cutometer®).

**Methods:**

Ten healthy participated in the study. Skin mechanical properties were measured at four sites: upper arm, lower arm, upper leg and lower leg on both sides in supine position using a non-invasive skin elasticity meter by two trained different raters. The measurements include quantitative indices of the maximal distensibility (R0), elasticity (R2, R5, R7), and viscoelasticity (R6). Intra- and inter- relative reliabilities were determined using the intraclass correlation coefficient (ICC) (1,1) and ICC (2,1) methods, respectively. The absolute reliability was assessed via the Bland-Altman analysis. Moreover, we evaluated the minimal detectable change at a 95% confidence level (MDC95).

**Results:**

At each site, the ICC (1,1) values were >0.90, and the ICC (2,1) values were >0.50. The Bland–Altman analysis did not reveal any fixed errors, and several sites and parameters have proportional errors.

**Conclusions:**

In this study, intra- and inter-reliabilities were measured at “excellent” and more than “moderate” levels, respectively. However, because some proportional errors were observed, the limits of reliability agreement should be considered when using the proposed methods. We believe that the results of this study can be applied to clinical research in field of rehabilitation treatment.

## Introduction

Skin is the largest organ of the human body and has mechanical properties such as stiffness, thickness, retraction, extension, pliability, elasticity, and viscosity. The mechanical properties of the skin are known to play a crucial role in functioning as a barrier to protect the body from the external environment and withstand mechanical stresses associated with physical activity and other factors [1,2]. Furthermore, the mechanical properties of the skin are affected by age, gender, diseases, and many other factors [3–5]. However, studies on skin-related structures and biomechanics remain limited.[6], Moreover, rehabilitation clinical practice depends on palpation, which is not an objective parameter, of changes in the mechanical properties of the skin. Therefore, it is worth investigating the mechanical properties of the skin.

The skin elasticity meter (Cutometer®; Courage & Khazaka Electronic GmbH, Cologne, Germany) is a non-invasive suction device developed to perform objective and quantitative measurements of the mechanical properties of the skin. Various methods (e.g. ultrasound) exist to objectively measure mechanical properties of the skin, and using the skin elasticity meter is one such option. The device operates via a load-controlled mechanism and uses an optical measurement system to track tissue utilized in the cosmetic industry [7,8] and in clinical setting to assessment various skin disease [9–12].

However, in many studies, aspects such as the limb position and measurement site have not been standardized. Although comparisons can be made within one study, it is generally difficult to compare results with those of other studies. To the best of our knowledge, no previous studies have compared the results of studies conducted using uniform measurement methods. Hence, the standard values and errors for each site measured by the skin elasticity meter have not been clearly defined.

Therefore, the purpose of this study is to examine the intra- and inter-reliabilities of the measurements performed on healthy skin using the skin elasticity meter in specific limb positions. A further direction of this study is also aimed at providing more information on the effects of rehabilitation treatment on patients with lymphedema, skin diseases, and postoperative orthopaedic surgery, based on the mechanical properties of their skin.

Measurement of the mechanical properties of the skin is of great interest in various fields. In clinical settings, measurement of the mechanical properties of the skin is an integral step in the characterization of both dermal aging and disease mechanisms and in the assessment of tissue-engineered skin replacements [13].

## Materials and methods

### Study design

The study was designed to examine the intra- and inter-rater reliabilities and absolute reliability of the measurements performed on healthy skin using the skin elasticity meter in specific limb positions. Therefore, this study is a cross-sectional observational study with a test-retest design (within-day and 1 week apart).

### Study participants

Ten healthy males participated in this study. It was confirmed that all the included participants did not suffer from any impairments, such as orthopaedic or neurosurgical disorders in both the upper and lower limbs. Measurements were performed for both the upper and lower limbs of the ten participants. In short, in the ten different participants, each of the two independent raters measured each of the 8 site areas in a total of 40 limbs.

The required sample size for the intra-rater correlation coefficient was calculated to provide the magnitude of the coefficient (*p*) 0.8, number of raters (*k*) 2, and a power of 80%, with a two-sided alpha level of 0.05. At least eight participants were required to reach statistically significant and clinically important differences based on the previous assumptions.

### Measurement of skin mechanical properties

We performed measurements using skin elasticity meter, a non-invasive suction device applied to quantitatively assess the mechanical properties of the skin.

A probe with a diameter of 2 mm was used in this study. The skin elasticity meter setting controlled a vacuum of 450 mbar on the skin surface for 3s, followed by 3s of normal pressure (Mode 1 setting).

**Figure 1.**
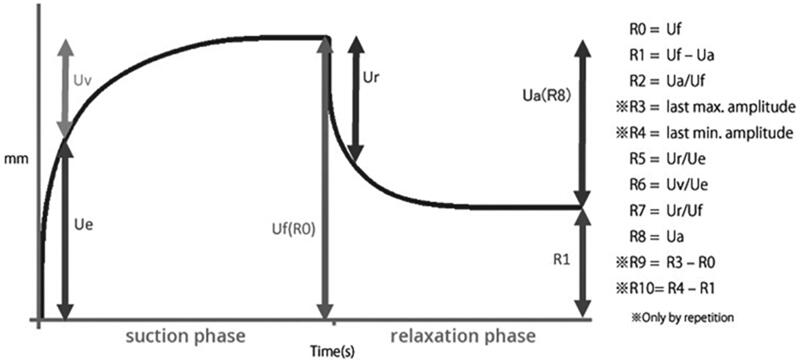
Skin elasticity meter graph and R parameters.

The parameters are expressed as absolute (Ua, Ue, Uf, and Uv) or relative parameters (R0-R9). Among them, the most commonly reported parameters include R0, R2, R5, R6, and R7. In accordance with these reports, these parameters were also measured in this study. R0, R2, R5, R6, and R7 indicate distensibility, gross elasticity, net elasticity, viscoelasticity, and biological elasticity, respectively (Figure 1).

### Measurement procedure

**Figure 2.**
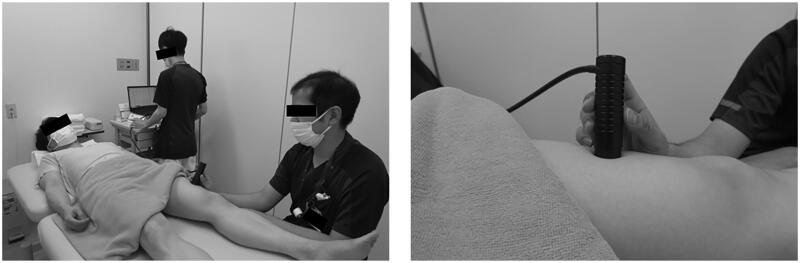
Skin elasticity meter set-up for measuring the mechanical properties of the skin. (a) The participant is depicted to be in a supine position while the rater holds the skin elasticity meter probe against the anterior surface of the left thigh. Measurements ware performed using this method at a total eight sites on the upper and lower limbs. (b) The exact position of the probe is illustrated.

Both independent raters (rater A and B) received training in the use of skin elasticity meter and standardization of the measurement procedure prior to data collection. During the experiments, each participant lay on the treatment bed in a relaxed supine position with both elbow and knee extended, and they maintained the posture throughout the skin elasticity meter measurement (Figure 2). The mechanical properties of the skin were measured at the upper arms, lower arms, upper legs, and lower legs. The four standardized measurement sites were defined as follows: (1) upper arm: 10 cm proximal to the elbow crease along the line parallel to the arm axis from the midpoint of the medial and lateral epicondyles, (2) lower arm: 5 cm distal to the elbow crease along the line parallel to the arm axis from the midpoint of the medial and lateral epicondyles, (3) upper leg: 12 cm proximal to the knee crease along the line parallel to the thigh axis from the midpoint of patella, and (4) lower leg: the largest part of the lower leg circumference, approximately 3 cm outside the tibial ridge. These measurement sites were partially modified in accordance with the Japanese lymphedema guidelines [14]. Measurements were performed three times at each site and the average values were used. Furthermore, each rater was blinded to the previous measurements to avoid any bias. The measurements were performed at room temperature and humidity, maintained at 23 ± 2 °C and 50–60%, respectively.

### Statistical analyses

Demographic data are presented as the mean ± standard deviation. Comparisons between the first and second offset measurement values of rater A were performed using a paired *t*-test.

Intra- and inter-rater reliabilities were evaluated using intraclass correlation coefficients (ICC) with 95% confidence interval (CI). Intra- and inter-reliabilities were determined using the ICC (1,1) and ICC (2,1) methods, respectively. ICC values were interpreted as follows: <0.5, poor reliability; 0.5–0.75, moderate reliability; 0.75–0.90, good reliability; >0.90, excellent reliability [15]. Precision was calculated using the standard error of measurement (SEM).

Absolute reliability was evaluated using Bland-Altman analysis to obtain intra- and inter-rater reliabilities [16]. Bland–Altman plots and limits of agreement were used to analyze the repeatability of a single measurement method and to compare measurements between the two raters. Moreover, we calculated a 95% confidence interval for the mean difference between the two measured values and determined that a fixed error was present if the resulting interval did not contain zero. Furthermore, we inspected the existence of a proportional error by calculating the *T*-value, and the level of significance was 5%, indicating the existence of a proportional error.

The limit of agreement was determined when fixed and/or proportional errors were present, the limit of agreement (LOA) was obtained. Additionally, if a random error was present without any systematic errors, we calculated the minimal detectable change at a 95% confidence level (MDC95). All statistical analyses were performed using the R (4.2.3) software, with the level of significance set at 5%.

### Ethical statement

The present study was carried out in accordance with the principles of the Declaration of Helsinki regarding investigations including humans and was approved by the ethics committee of the Osaka International Cancer Institute (approval number 22196) and Morinomiya University of Medical Science (approval number 2023-049). Each participant received an explanation of the study’s purpose and provided written informed consent prior to inclusion.

## Results

### Participants characteristics

The baseline characteristics of the ten study participants are presented in Table 1. The age, height, weight and body mass index (BMI) of the participants were 33.7 ± 7.9 years, 173.2 ± 6.1 cm, 65.0 ± 6.9 kg, and 21.7 ± 2.0 kg/m^2^, respectively.

**Table 1.** Participant characteristics.

Characteristic	Mean (*n* = 10)
Age, years	33.7 ± 7.9
Height, cm	173.2 ± 6.1
Weight, kg	65.0 ± 6.9
BMI, kg/m^2^	21.7 ± 2.0

Data are given as mean ± standard deviation. BMI: body mass index.

### Measurement values

The measurement values of the limbs obtained by the two independent raters are presented in Table 2. There were no significant differences in the offset values at each site between the first and second measurements of rater A.

**Table 2.** Measurement values (from above (a) upper arm, (b) lower arm, (c) upper leg, (d) lower leg).

Parameters	Rater A (once)	Rater A (twice)	Rater B
a. Upper arm			
Offset	0.328 ± 0.033	0.326 ± 0.031	0.330 ± 0.021
R0 (mm)	0.421 ± 0.078	0.419 ± 0.076	0.421 ± 0.071
R2 (%)	90.1 ± 4.1	90.5 ± 4.3	89.3 ± 4.1
R5 (%)	103.0 ± 8.6	103.7 ± 8.8	99.5 ± 8.4
R6 (%)	38.0 ± 10.7	37.6 ± 10.3	39.2 ± 13.0
R7 (%)	72.6 ± 6.4	73.5 ± 6.1	74.0 ± 4.4
b. Lower arm			
Offset	0.326 ± 0.028	0.327 ± 0.027	0.319 ± 0.028
R0 (mm)	0.328 ± 0.048	0.330 ± 0.048	0.341 ± 0.043
R2 (%)	88.5 ± 4.1	88.4 ± 3.8	89.3 ± 3.2
R5 (%)	111.7 ± 8.3	111.79 ± 8.4	111.2 ± 9.5
R6 (%)	47.4 ± 11.9	48.8 ± 12.6	49.1 ± 12.7
R7 (%)	75.7 ± 6.6	76.1 ± 5.6	75.6 ± 3.9
c. Upper leg			
Offset	0.318 ± 0.037	0.314 ± 0.036	0.300 ± 0.030
R0 (mm)	0.316 ± 0.078	0.325 ± 0.073	0.325 ± 0.065
R2 (%)	92.2 ± 4.2	91.9 ± 3.9	89.6 ± 3.9
R5 (%)	97.4 ± 11.6	99.1 ± 11.6	100.1 ± 13.1
R6 (%)	35.6 ± 10.8	35.5 ± 9.0	36.1 ± 7.5
R7 (%)	76.4 ± 8.5	76.0 ± 8.4	73.5 ± 9.3
d. Lower leg			
Offset	0.302 ± 0.030	0.300 ± 0.030	0.291 ± 0.029
R0 (mm)	0.20 ± 0.07	0.20 ± 0.07	0.22 ± 0.07
R2 (%)	89.5 ± 9.3	90.1 ± 9.0	89.8 ± 7.6
R5 (%)	107.2 ± 15.7	107.4 ± 14.8	110.3 ± 16.1
R6 (%)	59.3 ± 15.3	59.0 ± 13.9	57.5 ± 13.0
R7 (%)	72.6 ± 9.6	72.9 ± 9.9	71.2 ± 10.0

### Intra-rater reliabilities

The results of the intra-rater reliability analysis are presented in Table 3. The statistical analysis show excellent intra-rater reliability (>0.90) and 95% CI are 0.78–0.99 for all parameters in all sites. The SEM values are small (0.004–4.22) for each measurement parameter. Based on the results of the Bland–Altman test, there were no fixed errors, a proportional error was present only in the R7 parameter of the upper limb. The MDC95 values are 0.01–0.05 mm for R0 parameter and 2.02–11.69% for parameters R2–R7.

**Table 3.** Intra-rater reliability of measuring the mechanical properties of the skin using a skin elasticity meter.

					Absolute reliability							
		Relative reliability			Fixed error			Proportional error				MDC_95_
		ICC (1,1)	95% CI	SEM	95% CI	Result	LOA	*T* value	*p*	Result	LOA	
Upper arm	R0 (mm)	0.99	0.97–0.99	0.007	−0.03 ∼ 0.01	No	―	1.14	0.27	No	―	0.02
	R2 (%)	0.91	0.78–0.96	1.30	−2.46 ∼ 1.75	No	―	−0.31	0.76	No	―	3.59
	R5 (%)	0.93	0.82–0.97	2.40	−4.57 ∼ 3.22	No	―	−0.25	0.80	No	―	6.65
	R6 (%)	0.96	0.96–0.98	2.17	−3.14 ∼ 3.92	No	―	0.53	0.60	No	―	6.01
	R7 (%)	0.96	0.90–0.98	1.22	−2.60 ∼ 1.37	No	―	0.70	0.49	No	―	3.38
Lower arm	R0 (mm)	0.99	0.97–0.99	0.004	−0.01 ∼ 0.01	No	―	0.16	0.88	No	―	0.01
	R2 (%)	0.96	0.89–0.98	0.85	−1.29 ∼ 1.46	No	―	0.99	0.33	No	―	2.34
	R5 (%)	0.95	0.89–0.98	1.82	−3.02 ∼ 2.90	No	―	−0.21	0.84	No	―	5.05
	R6 (%)	0.95	0.88–0.98	2.63	−5.67 ∼ 2.90	No	―	−0.84	0.41	No	―	7.30
	R7 (%)	0.97	0.93–0.99	1.07	−1.84 ∼ 1.64	No	―	3.51	< 0.01	Yes	−2.72 ∼ 2.26	―
upper leg	R0 (mm)	0.93	0.84–0.97	0.02	−0.04 ∼ 0.02	No	―	0.84	0.41	No	―	0.05
	R2 (%)	0.97	0.92–0.99	0.73	−0.91 ∼ 1.47	No	―	1.18	0.25	No	―	2.02
	R5 (%)	0.93	0.83–0.97	2.97	−6.52 ∼ 3.15	No	―	0.05	0.96	No	―	8.24
	R6 (%)	0.92	0.80–0.97	2.96	−4.74 ∼ 4.89	No	―	2.17	0.07	No	―	8.22
	R7 (%)	0.97	0.92–0.99	1.59	−2.20 ∼ 2.98	No	―	0.08	0.94	No	―	4.41
lower leg	R0 (mm)	1.00	0.99–.099	0.004	−0.008 ∼ 0.005	No	―	0.36	0.73	No	―	0.01
	R2 (%)	0.98	0.95–0.99	1.33	−2.74 ∼ 1.58	No	―	0.74	0.47	No	―	3.69
	R5 (%)	0.97	0.92–0.99	2.74	−4.66 ∼ 4.25	No	―	1.03	0.31	No	―	7.59
	R6 (%)	0.92	0.81–0.97	4.22	−6.56 ∼ 7.14	No	―	0.99	0.34	No	―	11.69
	R7 (%)	0.98	0.95–0.99	1.42	−2.56 ∼ 2.05	No	―	−0.63	0.54	No	―	3.94

### Inter-rater reliabilities

The results of the inter-rater reliability analysis are presented in Table 4. The statistical analysis revealed moderate to excellent inter-rater reliability (>0.50) and 95% CI were 0.03 to 0.98 for all parameters in all sites. The SEM values were greater than the intra-rater reliability across each measurement parameter (0.02–10.26). Based on the results of Bland-Altman, no fixed errors were present, a proportional error was present in the R7 parameter for the upper and lower arms and the R2 parameter for the lower leg. The MDC95 values were 0.05–0.12 mm for R0 parameter and 5.61–28.45% for parameters R2–R7.

**Table 4.** Inter-rater reliability of measuring the mechanical properties of the skin using a skin elasticity meter.

					Absolute reliability							
		Relative reliability			Fixed error			Proportional error				MDC 95
		ICC (2,1)	95% CI	SEM	95% CI	Result	LOA	*T* value	*p*	Result	LOA	
Upper arm	R0 (mm)	0.80	0.55–0.91	0.03	−0.06 ∼ 0.05	No	―	0.41	0.69	No	―	0.09
	R2 (%)	0.74	0.46–0.89	2.02	−2.10 ∼ 4.47	No	―	0.32	0.75	No	―	5.61
	R5 (%)	0.92	0.81–0.97	2.50	−3.67 ∼ 4.47	No	―	0.50	0.62	No	―	6.93
	R6 (%)	0.73	0.45–0.89	6.08	−11.49 ∼ 8.27	No	―	−1.48	0.16	No	―	16.85
	R7 (%)	0.76	0.49–0.90	2.64	−4.87 ∼ 3.73	No	―	2.27	0.04	Yes	−7.05 ∼ 5.18	―
Lower arm	R0 (mm)	0.80	0.56–0.91	0.02	−0.04 ∼ 0.02	No	―	0.78	0.44	No	―	0.05
	R2 (%)	0.57	0.19–0.80	2.30	−4.60 ∼ 2.87	No	―	0.90	0.38	No	―	6.36
	R5 (%)	0.73	0.44–0.89	4.71	−7.06 ∼ 8.26	No	―	−0.79	0.44	No	―	13.07
	R6 (%)	0.93	0.83–0.97	3.43	−5.94 ∼ 5.22	No	―	−0.09	0.93	No	―	9.52
	R7 (%)	0.73	0.43–0.88	2.54	−3.94 ∼ 4.32	No	―	2.50	0.02	Yes	−5.17 ∼ 5.41	―
upper leg	R0 (mm)	0.60	0.22–0.82	0.04	−0.07 ∼ 0.07	No	―	0.57	0.58	No	―	0.12
	R2 (%)	0.53	0.03–0.73	2.73	−2.14 ∼ 6.75	No	―	0.04	0.97	No	―	7.58
	R5 (%)	0.77	0.51–0.90	6.00	−10.76 ∼ 8.75	No	―	−0.85	0.41	No	―	16.64
	R6 (%)	0.83	0.62–0.93	3.46	−6.23 ∼ 5.02	No	―	1.40	0.18	No	―	9.59
	R7 (%)	0.54	0.16–0.79	5.94	−7.13 ∼ 12.16	No	―	−0.49	0.63	No	―	16.45
lower leg	R0 (mm)	0.66	0.33–0.85	0.04	−0.08 ∼ 0.04	No	―	−0.05	0.60	No	―	0.11
	R2 (%)	0.96	0.90–0.98	1.75	−4.526 ∼ 5.166	No	―	2.86	0.01	Yes	−2.80 ∼ 3.27	―
	R5 (%)	0.56	0.18–0.80	10.26	−19.60 ∼ 13.78	No	―	−0.44	0.67	No	―	28.45
	R6 (%)	0.96	0.89–0.98	2.73	−2.96 ∼ 5.92	No	―	1.08	0.29	No	―	7.57
	R7 (%)	0.90	0.76–0.96	3.01	−3.25 ∼ 6.54	No	―	−0.08	0.94	No	―	8.34

## Discussion

In this study, we demonstrated high intra- and inter-reliabilities in measuring the mechanical properties of the skin of healthy participants using a skin elasticity meter. Several studies have previously reported that the reliability of measurement using the skin elasticity meter is acceptable (ICC >0.75) for patients with burns [12,17], scars [18,19], systemic sclerosis [20], and healthy individuals [21]. However, these previous studies did not use standardized measurement methods. Therefore, little information is understood regarding reference values and errors at these sites. The novelty of this study is that it is the first to demonstrate the measurement values, reliability, and errors of skin mechanical properties at four limb sites, which will be useful for future measurement studies.

Among the results of the present measurement values, the R0 parameter for each site showed good intra- and inter-rater reliabilities and no systematic errors. This result is consistent with those of a previous study [22–24]. The R0 parameter correlates with the skin pliability/firmness and represents the passive behaviour of the skin toward the suction force. This parameter was measured from the highest point of the amplitude at the end of the suction phase to the baseline reading [25]. Therefore, the R0 parameter can be reliably measured without being affected by the rater or probe during the measurement by training the offset values to be uniform. However, parameters R2, R5, R6, and R7 are presented using the formulas of the point ratio and are not measured in units of length. Consequently, these parameters may hold less utility given the inherent difficulty in establishing standards across a heterogeneous population of participants. However, these parameters may be useful for understanding the mechanical properties of the individual skin, and it is necessary to consider their use. Consequently, we conclude that R0 is an essential output parameter for clinical applications of the present measurement method.

However, when R0 was included, the inter-rater reliability values were lower than the intra-rater reliability values. This result is consistent with those of several previous ICC studies [25]. In previous studies, it affected the measured values when using skin elasticity meter, such as the operational skill of the rater, fixation of the probe [26,27], and the participants’ movement [28]. While these causes are prominent among inter-raters, they can be made constant to some extent within the intra-raters. Therefore, when using the skin elasticity meter in longitudinal studies, we believe that it is best to perform the measurements with the same rater to maintain a high level of reliability.

In addition, proportional errors were confirmed for several site parameters. A proportional error grows in proportion to the magnitude of the true value. In this study, the parameters R2 and R7, for which a proportional error was present, are the amplitudes after the value of R0 in the suction phase obtained and the percentage of delayed (R2) and immediate (R7) skin recovery. As noted above, no systematic error was present in R0; therefore, it was assumed that a specific aspect in the skin recovery process caused this error. Although we were unable to identify a clear reason for the error. In accordance with previous studies, the probe operation and skin characteristics of the participants were considered. In summary, for the parameters at the site where proportional error was present, the true difference must be determined when measuring with reference to the LOA set in this study. Moreover, in this study, the MDC95 was calculated for the parameters of sites where both fixed and proportional errors were absent. MDC95 indicates the limit of the two measurement values. If the obtained value is within MDC95, it was considered to be due to a measurement error. However, a true change occurs if it is higher than MDC95. The MDC95 obtained in this study is a useful indicator for determining whether clinically effective changes have occurred.

This study has several limitations. First, there is a potential for selection bias because we only included young males, and the results cannot be generalized to all age and sex groups. In other words, to examine the reliability of this method across a wide range of ages and different genders, it is necessary to study the method based on this methodology. Second, we were unable to examine reliability in patients with skin thickness, stiffness, elasticity, and viscosity disorders. However, this study clearly demonstrated the reliability of this measurement method. Therefore, we believe that this measurement method has study applications (e.g. disease and age). In the future, our results should be confirmed in actual patients.

## Conclusion

We conclude that the intra- and inter- rater reliabilities were sufficient in the measurement of the mechanical properties of the skin in the limbs of healthy participants using the skin elasticity meter in specific limb positions. Furthermore, the error and MDC95 of the measurements of the mechanical properties of the skin presented in this study are useful as a basis for interpreting the measurements. The result, the measurement of skin mechanical properties using the skin elasticity meter in specific limb positions could be considered for application in clinical research in rehabilitation.

## Data Availability

Data are available upon reasonable request from corresponding author.
